# Translesion DNA Polymerases and Cancer

**DOI:** 10.3389/fgene.2012.00174

**Published:** 2012-09-06

**Authors:** Nick M. Makridakis, Juergen K. V. Reichardt

**Affiliations:** ^1^Tulane Cancer Center and Department of Epidemiology, Tulane UniversityNew Orleans, LA, USA; ^2^School of Pharmacy and Molecular Sciences, James Cook UniversityTownsville, QLD, Australia

**Keywords:** error-prone, DNA damage, instability, pharmacogenetics

## Abstract

DNA repair has been regarded as an important barrier to carcinogenesis. The newly discovered field of translesion synthesis (TLS) has made it apparent that mammalian cells need distinct polymerases to efficiently and accurately bypass DNA lesions. Perturbation of TLS polymerase activity by mutation, loss of expression, etc. is expected to result in the accumulation of mutations in cells exposed to specific carcinogens. Furthermore, several TLS polymerases can modulate cellular sensitivity to chemotherapeutic agents. TLS genes and TLS gene variations may thus be attractive pharmacologic and/or pharmacogenetic targets. We review herein current data with regards to the potential contribution of the primary TLS polymerase genes to cancer, their interaction with pharmacologic agents, and identify areas of interest for further research.

## Translesion DNA Synthesis and Cancer

Tumors need somatic mutations in order to evolve; yet they also need efficient DNA replication. Reducing the fidelity of a human polymerase without affecting its activity [as in the case of mutations affecting the exonuclease (proofreading) domain of the replicative human DNA polymerase delta; da Costa et al., 1995], may be one way that tumors achieve both. Another way may involve the recently discovered, error-prone (EP) DNA polymerases (Kunkel et al., 2003). These polymerases are distributive enzymes with very low fidelity when copying undamaged DNA (Kunkel et al., 2003) yet they copy damaged DNA [in a process called translesion synthesis (TLS)] with much higher efficiency compared to the replicative DNA enzymes (Goodman, 2002; Pages and Fuchs, 2002). These findings have led to a model (Goodman, 2002; Pages and Fuchs, 2002) proposing that during DNA replication the replicative DNA polymerase gets stalled at sites of DNA damage and it is replaced by an EP polymerase. TLS operates in addition to other, known pathways of DNA repair (reviewed e.g., by Sancar et al., 2004).

Error-prone polymerases lack proofreading activity and can copy through a specific lesion in either a mutagenic or an accurate manner, depending on the particular lesion, the polymerase used and the DNA sequence context (Pages and Fuchs, 2002). For example, the EP polymerase eta (Pol η) performs accurate TLS *in vitro* over a thymine–thymine (TT) cyclobutane dimer, a cis-platinum adduct, an acetyl aminofluorene (AAF) adduct, or 8-oxo guanine yet it results in mutagenic TLS on benzopyrene-N2-dGuanine (Goodman, 2002). In contrast, polymerase kappa (Pol κ) can perform accurate TLS on benzopyrene-N2-dGuanine adducts (Ohashi et al., 2000) yet it results in mutagenic TLS at 8-oxo guanine lesions (Zhang et al., 2000). Thus, a somatic inactivating mutation (genetic contributor) in, e.g., *POLK* can result in subsequent mutagenesis of specific genes (based on sequence context), and eventually cancer, in cells exposed to benzopyrene (environmental contributor), due to mutagenic TLS repair by the “wrong” polymerase (e.g., Pol η). Different combinations of EP polymerases/lesions/sequence contexts can result in distinct sets of mutated genes and thus specific cancers, without necessarily affecting DNA synthesis efficiency.

Cancer-inducing mutagenesis resulting from the use of the wrong EP polymerase has been proposed in order to explain the phenotype of Xeroderma pigmentosum-variant (XPV) patients (Kanouche et al., 2002; Kunkel et al., 2003). XPV is a rare inherited human disorder characterized by increased incidence of sunlight-induced skin cancers that is caused by inactivating mutations of *POLH* (Masutani et al., 1999). In the absence of Pol η. Activity, XPV cells cannot perform accurate bypass of ultraviolet (UV)-light induced TT-dimers, and the resulting mutations at TT sites are thought to cause skin cancer (e.g., Kanouche et al., 2002; Kunkel et al., 2003; Stary et al., 2003).

## POLB

**Figure 1:**
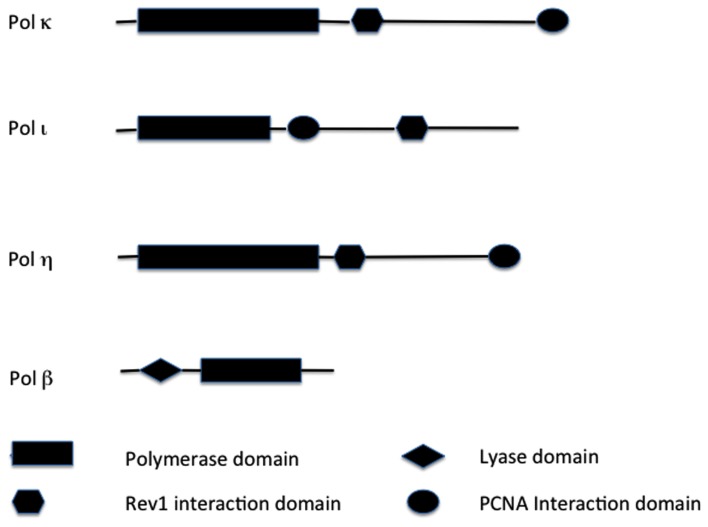
**Structural comparisons of  TLS enzymes**. Major functional domains are indicated. Not drawn to scale. Key: PCNA, proliferating cell nuclear antigen.

Human polymerase beta (Pol β) is a monomeric protein of 335 residues (Table 1; Figure 1) that is essential for base excision repair (BER; Goodman, 2002). BER is one of the major pathways of DNA repair that removes oxidized and alkylated bases from DNA (Friedberg, 2003). Pol β is not a classic EP polymerase, yet it causes 67-times more errors than mammalian Pol δ (Table 1; Kunkel et al., 2003). Pol β often bypasses a DNA lesion by insertion of a complementary nucleotide to an adjacent downstream template site, resulting in both deletion and substitution errors (Efrati et al., 1997). Pol β is also involved in meiotic recombination (Kidane et al., 2010) and is critical for genomic stability in germ cells (Kidane et al., 2011). Targeted disruption of Pol β in mice resulted in neonatal lethality (due to respiratory failure) growth retardation and apoptotic cell death in the developing nervous system (Sugo et al., 2000), suggesting a role for Pol β in neurogenesis.

**Table 1:** **Human translesion synthesis DNA polymerase genes**.

Gene	Chromosomal location	Gene structure (exons)	Protein size (amino acids)	Error rate (×10^−5^)
*POLB*	8p11	14	335	67
*POLH*	6p21	11	713	3500
*POLK*	5q13	15	870	580
*POLI*	18q21	10	740	24300

*Error rate refers to the average single base substitution rate per incorporated nucleotide during DNA synthesis *in vitro*, taken from Kunkel et al. (2003). By comparison, the replicative polymerase Pol δ has an error rate of 1 × 10^−5^ (Kunkel et al., 2003)*.

Pol β transcription and protein levels are increased in many cancer cells (e.g., prostate, breast, colon, ovarian, leukemia; Scanlon et al., 1989; Louat et al., 2001) and overexpression of Pol β results in aneuploidy and tumorigenesis in nude immunodeficient mice (Bergoglio et al., 2002). Moreover, *pol β* heterozygous mice exhibit increased single-stranded DNA breaks, chromosomal aberrations, and mutagenicity compared to normal animals (Cabelof et al., 2003). Thus both higher and lower Pol β activity can result in increased mutagenesis *in vivo*. These studies suggest that accurate regulation of Pol β expression may be essential *in vivo*. Deregulated Pol β may cause chromosomal instability either by competing with replicative polymerases or by TLS over DNA lesions. Indeed, Pol β exhibits a very low fidelity in DNA synthesis opposite both 8-oxo-G and 1,2-dihydro-2-oxoadenine (2-OH-A) lesions (Frechet et al., 2002). These data suggest that Pol β may be an important factor in causing some of the genetic changes associated with cancer.

*POLB* has been called the “platinum resistance gene” (Iwatsuki et al., 2009) due to the fact that reduced *POLB* expression in colorectal cancer cells results in susceptibility to cisplatin (Iwatsuki et al., 2009). In contrast, the same experiment performed in mouse embryonic fibroblasts results in sensitivity to oxaliplatin, but not cisplatin (Yang et al., 2010). These differences may be due to tissue- and/or development- specific factors. Higher Pol β expression also correlated with poorer colorectal cancer prognosis (Iwatsuki et al., 2009), underscoring the significance of this gene for chemotherapy.

### Germline single nucleotide polymorphisms of POLB

Seven non-synonymous DNA substitutions of *POLB* have been described in dbSNP in normal individuals^1^. These single-nucleotide polymorphisms (SNPs) display minor allele frequencies (MAF) of 2–10%. Four of these variants have been validated, including three common SNPs (i.e., SNPs with MAF > 1%; Table 2). Two of these variants have been characterized biochemically thus far. The p.R137Q variant (refers to rs12678588; Table 2) displayed abnormal BER activity through both decreased catalytic activity and aberrant interaction with PCNA (Guo et al., 2009). The p.P242R variant (refers to rs3136797; Table 2) displayed altered fidelity of DNA synthesis for all possible dNTP misincorporations across a single-nucleotide gapped template (An et al., 2011). Given the known mechanistic effects of the studied missense variants of Pol β (e.g., Guo et al., 2009; An et al., 2011), it is likely that some of the yet uncharacterized non-synonymous nucleotide substitutions of Pol β affect polymerase function.

**Table 2:** **Genetic variants of translesion synthesis DNA polymerase genes**.

Gene	Common validated ns-SNPs*(MAF; population)	Somatic mutations(freq; tissue)	Citation**
*POLB*	rs12678588 (0.04; MEX)	many (15–75%; many tissues)	Starcevic et al. (2004), Makridakis et al. (2009)
	rs56121607 (0.02; CH)		
	rs3136797 (0.02; AA)		
*POLH*	rs2307456 (0.03; MEX)	7 (1.5–12%; melanoma, breast, prostate)	Makridakis et al. (2009), Di Lucca et al. (2009), Sjöblom et al. (2006)
	rs35675573 (0.02;YRI)		
	rs9296419 (0.1; CH)		
	rs9333554 (0.07;YRI)		
	rs9333555 (0.04; GIH)		
	rs6941583 (0.411; LWK)		
*POLK*	rs35257416 (0.03; AA)	6 (1–100%; prostate, ovarian, kidney)	Makridakis et al. (2009), Cancer Genome Atlas Research Network (2011), Dalgliesh et al. (2010)
	rs3822587 (0.05; CH)		
*POLI*	rs3218786 (0.04; CAU)	3 (1–33%; ovarian, kidney)	Cancer Genome Atlas Research Network (2011), Dalgliesh et al. (2010)
	rs8305 (0.380; CAU)		

*Common = minor allele frequency > 1% (dbSNP Build 134).

**Refers to somatic mutations.

*Key: MAF, the highest minor allele frequency reported in dbSNP for any racial/ethnic group; freq, % patients with a somatic polymerase mutation; AA, African American; CAU, Caucasian American; CH, Han Chinese; GIH, Gujarati Indians; LWK, Luhya in Webuye, Kenya; MEX, Mexican American; YRI, Yoruba*.

### Somatic mutations of POLB in tumors

Table 2 shows that somatic Pol β variants are present in 15–75% of tumors analyzed to date (e.g., Starcevic et al., 2004; Makridakis et al., 2009). Functional analysis has implicated many of these variants in cancer etiology and/or progression. Specifically, the p.K27N and the triple mutant (p.P261L/T292A/I298T) variants reduced catalytic efficiency, while the p.E123K, p.E232K, p.P242R, p.E216K, and p.M236L variants altered the fidelity of DNA synthesis at steady-state conditions (An et al., 2011). Furthermore, overexpression of either the p.K289M or p.I260M Pol β variants in mouse fibroblasts, resulted in cellular transformation (Dalal et al., 2005; Sweasy et al., 2005). The finding that the transformed phenotype remained after expression of these variants had ceased in these cells, suggests that transformation occurred after further mutations induced by the Pol β variant, thereby supporting the mutagenic model for cancer etiology proposed above. Given the high frequency of functional somatic *POLB* mutations in tumors, and the correlation of *POLB* expression with survival and drug susceptibility, further pharmacologic and pharmacogenetic studies will be highly beneficial.

## POLH

Pol η belongs to the Y polymerase family (which includes REV1, Pol κ, and Pol ℩; Figure 1). In humans, Pol η is encoded by the *POLH* gene, also known as XPV gene, because it was found mutated in a fraction of patients suffering from XP disease that did not carry mutations in nucleotide excision repair (NER) genes (see above; Johnson et al., 1999a; Masutani et al., 1999). *POLH* lies on chromosomal band 6p21 (Table 1). Loss of Pol η results in a reduced ability to copy DNA containing a very common form of sunlight-induced damage, TT-dimers (Johnson et al., 1999b; Masutani et al., 1999). Pol η can bypass these lesions with high accuracy and processivity, but is less processive and more inaccurate on undamaged DNA templates (Matsuda et al., 2001; Washington et al., 2001). In addition to pyrimidine dimmers, Pol η can efficiently bypass a wide variety of DNA lesions, in both EP and error-free manner, including mitomycin C adducts (Zheng et al., 2003), and cisplatin adducts (Albertella et al., 2005; Alt et al., 2007).

Pol η shows higher catalytic efficiency than Pol β when bypassing cisplatin adducts (Chaney et al., 2005). Furthermore, Pol η downregulation results in increased sensitivity to cisplatin (Chen et al., 2006). Not surprisingly, high *POLH* expression associated with significantly shorter survival, in a group of platinum treated non-small cell lung cancer patients (Ceppi et al., 2009). Moreover, the kinase inhibitor LY294002 enhanced the killing of cells deficient in Pol η, synergistically with the chemotherapeutic agent doxorubicin (Moraes et al., 2012) underscoring the ability of Pol η to regulate cellular response to these agents.

Pol η can also modulate cellular response to anticancer nucleoside analogs. Indeed, Pol η efficiently extends from both arabinosylcytocine (AraC) and gemcitabine containing 3′ DNA ends, and reduces cellular sensitivity to both of these nucleoside analogs (Chen et al., 2006). These data make Pol η an attractive candidate for pharmacogenetic studies.

### Germline SNPs of POLH

Many missense skin cancer-predisposing Pol η mutations have been identified in XPV patients (reviewed in Gratchev et al., 2003). In addition, 37 non-synonymous *POLH* substitutions have been described in dbSNP in normal individuals^2^. These SNPs display MAF of 0.3–11.4%. Twelve of these SNPs have been validated, including common SNPs (Table 2). One of the common non-synonymous *POLH* variants, rs9333555 (Table 2) was found to be significantly associated with melanoma risk (OR = 1.84; Di Lucca et al., 2009). This finding was confirmed in a meta-analysis (Di Lucca et al., 2009). Thus, other types of skin cancer are also good candidates for the identification of *POLH* cancer-predisposing alleles.

### Somatic mutations of POLH in tumors

No somatic mutations of Pol η were found in two screens of 15 and 17 basal/squamous cell carcinomas of the skin (Glick et al., 2006; Flanagan et al., 2007). However, three missense *POLH* mutations were identified in three out of 201 melanoma patients, and these mutations were absent in 176 healthy controls (Di Lucca et al., 2009). These results warranty a more careful examination of *POLH* for somatic mutations in large numbers of skin tumors. Furthermore, biochemical analysis of the cancer-associated variants will be necessary in order to distinguish tumor “drivers” from mere passengers of tumor evolution. Interestingly, the same somatic *POLH* mutation, p.G153D, was identified by two independent studies, in a small subset (2–9%) of breast tumors^3^ (Sjöblom et al., 2006). By screening only one exon of the *POLH* gene, we identified three missense somatic mutations in two out of 26 prostate tumors (Makridakis et al., 2009). One of these three Pol η mutations that we identified is in glycine-263 (Makridakis et al., 2009). Homozygous missense mutation of Pol η glycine-263 has been reported in an XPV patient (Broughton et al., 2002). These data suggest that Pol η may play a role in tumor progression in different types of cancer. Given the protective role of Pol η with various types of chemotherapeutic drugs (see above), further molecular genetic and pharmacogenetic studies are warrantied.

## POLK

Pol κ (Figure 1) is a Y polymerase family enzyme encoded by the *POLK* gene, which lies on chromosomal band 5q13 in humans (Table 1). Pol κ is the human polymerase mostly similar to the E. coli polymerase pol IV/DinB (Hubscher et al., 2002). Pol κ can bypass benzo[a]pyrene diol epoxide (BPDE) adducts on the N2 of guanine in an error-free manner (Rechkoblit et al., 2002; Suzuki et al., 2002). Pol κ can also efficiently bypass AAF-guanine and ethenodeoxy-adenosine adducts, but in an EP manner (Goodman, 2002). Pol κ expression was diminished in rat mammary carcinoma cell lines and primary mammary carcinomas in comparison to that of the normal tissues (Pan et al., 2005). In contrast, Pol κ is overexpressed in lung cancer (O-Wang et al., 2001). This discrepancy of differential expression in distinct types of tumors, may be an adaptation to different mutagen and/or adduct concentrations in distinct human tissues.

Mouse embryonic stem cells genetically depleted of Pol κ are highly sensitive to both killing and mutagenesis induced by benzopyrene, but not to UV-light or X-rays (Ogi et al., 2002). Expression of the mouse *pol κ* gene is under the control of the aryl hydrocarbon receptor, an important factor for the metabolic activation of benzopyrene to BPDE in mammalian cells (Ogi et al., 2001). These results suggest that Pol k may function *in vivo* in error-free TLS of lesions generated by polycyclic aromatic hydrocarbons (PAH), such as BPDE. Thus Pol κ inactivation may result in PAH-induced mutagenesis and cancer in cells exposed to PAH.

Furthermore, ectopic Pol κ overexpression induced aneuploidy, DNA strand breaks, and tumorigenesis in nude mice (Bavoux et al., 2005). The *POLK* gene was also shown to display loss of heterozygosity in non-squamous lung carcinomas compared to adjacent normal tissue (Bavoux et al., 2005). Thus, both the increase and decrease of Pol κ steady-state levels can promote a malignant phenotype.

In addition, Pol κ is up-regulated in mouse and human cells by a p53-dependent pathway, in response to various DNA-damaging agents (Velasco-Miguel et al., 2003), and also in response to functional loss of p53 (Wang et al., 2004). The involvement of both p53-dependent and p53-independent pathways for Pol κ upregulation suggests that Pol κ may play an important physiologic role in guarding genome stability.

Pol κ is able to bypass tamoxifen-derived DNA adducts more efficiently and accurately than Pol η (Yasui et al., 2006). Thus pharmacologic and/or pharmacogenetic studies with tamoxifen and tumor survival will be highly beneficial.

### Germline SNPs of POLK

Forty-seven non-synonymous *POLK* substitutions have been described in dbSNP in normal individuals^4^. These SNPs display MAF of 1.4–1.8%. Eighteen of these SNPs have been validated, including some common SNPs (Table 2). None of these SNPs have been evaluated biochemically for their effect on enzyme activity. However, analysis of 62 DNA repair genes identified two non-coding *POLK* SNPs that were significantly associated with lung cancer risk (Michiels et al., 2007). Moreover, eQTL analysis by the GTEx web browser^5^, indicates that the trans-eQTL SNP rs1828591 significantly associates with *POLK* gene expression in the liver (Schadt et al., 2008). These data underscore the potential significance of both coding and non-coding *POLK* SNPs in different types of cancer.

### Somatic mutations of POLK in tumors

Three distinct somatic missense *POLK* mutations have been reported so far in tumors by the Sanger Institute: one in ovarian cancer (p.R48I, in two out of two patients) and two in kidney cancer^6^ (p.F286S and p.M364L, in 1% of the patients). We also identified three somatic *POLK* mutations in prostate tumors, by screening two exons in 26 patients (Makridakis et al., 2009). One of these somatic substitutions changes the predicted invariant Adenine of intron five of *POLK* where the lariat forms (Makridakis et al., 2009), and thus may result in abnormal splicing. The other two somatic *POLK* mutations are missense (Makridakis et al., 2009). Both missense substitutions that we identified in *Pol κ* are in threonine-205 (Makridakis et al., 2009). The homologous residue in Pol η of threonine-205 of *Pol κ*, is threonine-122 (Boudsocq et al., 2002). Germline missense mutation of threonine-122 of human Pol η has been reported in an XPV patient (Broughton et al., 2002).

Thus, three of the somatic missense mutations that we identified in the Y-family polymerase genes *POLK* and *POLH* are in (or are in residues homologous to) residues of Pol η previously associated with XPV. This finding can be explained by two possibilities: (a) that these are mutational hotspots, or (b) that these mutations play a role in carcinogenesis. We favor the latter scenario. This explanation however generates an interesting question. XPV patients are born with a Pol η mutation. If the same mutation that is involved in prostate cancer etiology is also present in all of the cells of some XPV patients, then why do these patients get only skin cancer and not prostate (or other type of) cancer? The most likely explanation is different environmental exposure: XPV patients are inevitably exposed to sunlight, but not necessarily to prostate cancer-inducing environmental mutagens (the prostate gland is too deep inside the body to be affected by sunlight, and Pol η may be important for repairing DNA damage relevant to the prostate, such as oxidation damage). An alternative explanation though is that Pol η mutation maybe sufficient for skin carcinogenesis, but that an additional (presumably rare) molecular event is necessary for prostate carcinogenesis. Regardless, these data suggest that EP polymerase gene mutations may be common and prevalent in prostate cancer tissue.

## POLI

The human *POLI* gene lies in chromosomal band 18q21, and encodes for Polymerase  ι  (Figure 1). Pol  ι  is able to bypass many DNA lesions, such as pyrimidine dimers, oxidative damage, and PAH adducts, but it cannot extend after the intial incorporation (Vaisman and Woodgate, 2001; Frank et al., 2002; Wang et al., 2007). Thus, the utilization of Pol  ι  in TLS *in vivo* depends on an extension polymerase, such as Pol ζ (Johnson et al., 2000; Guo et al., 2001). The fidelity of Pol  ι  on undamaged DNA is generally low (Table 1) but very variable: error frequencies of 10^−4^ to 10^−5^ are observed opposite template A, 10^−1^ to 10^−2^ opposite templates G and C, while it prefers to incorporate the wrong nucleotide (mainly G) opposite template T (Zhang et al., 2000; Haracska et al., 2001; Tissier et al., 2000). Pol  ι  is able to perform BER *in vitro* (Bebenek et al., 2001). A possible explanation for this unusual preference of Pol  ι  opposite template T is that during *in vivo* BER, Pol  ι  would insert G opposite a template T that had been generated by deamination of a 5-methylated C, thus preventing C > T transitions at CpG islands.

Pol  ι  expression is elevated in breast cancer cells and correlates with a significant decrease in DNA synthesis fidelity (Yang et al., 2004). Furthermore, UV treatment of breast cancer cells additionally increased Pol  ι  expression, while a reduction in mutation frequency was shown after Pol  ι  was immunodepleted from nuclear extracts of the same breast cancer cells (Yang et al., 2004). These data suggest that Pol  ι  may play a role in breast cancer development and/or progression.

### Germline SNPs of POLI

Forty-nine non-synonymous *POLI* substitutions have been described in dbSNP in normal individuals^7^. These SNPs display MAF of 0.1–22%). Twenty-three of these SNPs have been validated, including common variants (Table 2). None of these SNPs have been evaluated biochemically for their effect on enzyme activity. However, a single-nucleotide polymorphism in *POLI* (rs8305), correlated with a significantly higher risk of both lung adenocarcinoma and squamous cell carcinoma (Sakiyama et al., 2005). Furthermore, another *POLI* SNP (rs3218786), was significantly associated with TMPRSS2-ERG fusion-positive prostate tumors (Luedeke et al., 2009).

### Somatic mutations of POLI in tumors

Three distinct somatic missense *POLI* mutations have been reported so far in tumors by the Sanger Institute: two in ovarian cancer (p.R28T, in one out of three patients; and p.K271E, in another patient) and one in kidney cancer^8^ (p.D306V, in 1% of the patients). These data suggest the need for more comprehensive analysis of tumor tissues.

## Other TLS Polymerases

In addition to the polymerases reviewed above, other low-fidelity DNA polymerases have TLS synthesis abilities (reviewed by Moon et al., 2007). The Y-family polymerase REV1 has been proposed to act as a scaffold for other TLS enzymes, such as Pol η and Pol κ (Tissier et al., 2004). Knockdown of *REV1* mRNA results in a significant reduction of UV-irradiation induced mutagenesis (Clark et al., 2003). Two distinct non-synonymous *REV1* SNPs have been associated with cervical cancer risk (He et al., 2008). Furthermore, six missense and one nonsense *REV1* substitutions have been reported by the Sanger Institute, in a minority of tumors^9^. These data imply the need for further interrogation of REV1 in cancer etiology and progression.

Members of the X family of DNA polymerases (such as Pol λ and Pol μ), can perform TLS with various efficiencies, depending on the lesion (Blanca et al., 2004; Moon et al., 2007). These polymerases display the second lowest replication fidelity among the six major DNA polymerase families (after the Y-family; Kunkel, 2004). Furthermore, Pol θ (a family A member), exhibits lower replication fidelity relative to the other A family members and has been suggested to participate in TLS *in vivo* (Arana et al., 2008). However, these non-Y-family polymerases have other primary functions *in vivo*, independent of TLS, such as non-homologous end joining (X family polymerases). Accordingly, we focused in this review mostly on the Y-family polymerases, which primarily function in lesion bypass (Friedberg et al., 2005).

## Conclusion

Translesion synthesis is the newest and less characterized pathway of DNA repair. It involves DNA polymerases that facilitate DNA replication (and thus cell division) by efficiently bypassing various DNA lesions in a relatively error-free manner. TLS polymerase gene expression, mutation, and regulation, is important for cancer etiology and treatment. Current pharmacologic data regarding the interactions of TLS polymerases with various mutagens and chemotherapeutic drugs underscore the significance of further pharmacologic and pharmacogenetic research.

## Conflict of Interest Statement

The authors declare that the research was conducted in the absence of any commercial or financial relationships that could be construed as a potential conflict of interest.

## Footnotes

^1^http://www.ncbi.nlm.nih.gov/SNP/snp_ref.cgi?locusId = 5423

^2^http://www.ncbi.nlm.nih.gov/SNP/snp_ref.cgi?locusId = 5429

^3^http://www.sanger.ac.uk/perl/genetics/CGP/cosmic?action = mut_summary&id = 13899

^4^http://www.ncbi.nlm.nih.gov/SNP/snp_ref.cgi?locusId = 51426

^5^http://www.ncbi.nlm.nih.gov/gtex/GTEX2/gtex.cgi

^6^http://www.sanger.ac.uk/perl/genetics/CGP/cosmic?action = bygene&ln = POLK&start = 1&end = 871&coords = AA:AA

^7^http://www.ncbi.nlm.nih.gov/SNP/snp_ref.cgi?locusId = 11201

^8^http://www.sanger.ac.uk/perl/genetics/CGP/cosmic?action = bygene&ln = POLI&start = 1&end = 716&coords = AA:AA

^9^http://www.sanger.ac.uk/perl/genetics/CGP/cosmic?action = bygene&ln = REV1&start = 1&end = 1252&coords = AA:AA
